# High Prevalence and Diversity of Hepatitis Viruses in Suspected Cases of Yellow Fever in the Democratic Republic of Congo

**DOI:** 10.1128/JCM.01847-16

**Published:** 2017-04-25

**Authors:** Sheila Makiala-Mandanda, Frédéric Le Gal, Nadine Ngwaka-Matsung, Steve Ahuka-Mundeke, Richard Onanga, Berthold Bivigou-Mboumba, Elisabeth Pukuta-Simbu, Athenaïs Gerber, Jessica L. Abbate, Dieudonné Mwamba, Nicolas Berthet, Eric Maurice Leroy, Jean-Jacques Muyembe-Tamfum, Pierre Becquart

**Affiliations:** a Centre International de Recherches Médicales de Franceville (CIRMF), Franceville, Gabon; b Département de Microbiologie, Cliniques Universitaires de Kinshasa (CUK), Kinshasa, Democratic Republic of the Congo; c Hôpital Avicenne, Laboratoire de Virologie, Centre National de Référence associé Hépatite Delta, Bobigny, France; d Institut National de Recherche Biomédicale (INRB), Kinshasa, Democratic Republic of the Congo; e Institut de recherche pour le développement (IRD), Montpellier, France; f Direction de lutte contre les maladies (DLM), Kinshasa, Democratic Republic of the Congo; g Centre National de Recherche Scientifique (CNRS) UMR 3569, Paris, France; h UMR UMMISCO (UMI 209 IRD-UPMC), Bondy, France; Memorial Sloan-Kettering Cancer Center

**Keywords:** DRC, hepatitis virus, yellow fever surveillance

## Abstract

The majority of patients with acute febrile jaundice (>95%) identified through a yellow fever surveillance program in the Democratic Republic of Congo (DRC) test negative for antibodies against yellow fever virus. However, no etiological investigation has ever been carried out on these patients. Here, we tested for hepatitis A (HAV), hepatitis B (HBV), hepatitis C (HCV), hepatitis D (HDV), and hepatitis E (HEV) viruses, all of which can cause acute febrile jaundice, in patients included in the yellow fever surveillance program in the DRC. On a total of 498 serum samples collected from suspected cases of yellow fever from January 2003 to January 2012, enzyme-linked immunosorbent assay (ELISA) techniques were used to screen for antibodies against HAV (IgM) and HEV (IgM) and for antigens and antibodies against HBV (HBsAg and anti-hepatitis B core protein [HBc] IgM, respectively), HCV, and HDV. Viral loads and genotypes were determined for HBV and HVD. Viral hepatitis serological markers were diagnosed in 218 (43.7%) patients. The seroprevalences were 16.7% for HAV, 24.6% for HBV, 2.3% for HCV, and 10.4% for HEV, and 26.1% of HBV-positive patients were also infected with HDV. Median viral loads were 4.19 × 10^5^ IU/ml for HBV (range, 769 to 9.82 × 10^9^ IU/ml) and 1.4 × 10^6^ IU/ml for HDV (range, 3.1 × 10^2^ to 2.9 × 10^8^ IU/ml). Genotypes A, E, and D of HBV and genotype 1 of HDV were detected. These high hepatitis prevalence rates highlight the necessity to include screening for hepatitis viruses in the yellow fever surveillance program in the DRC.

## INTRODUCTION

Yellow fever is one of the most lethal viral diseases transmitted by infected *Aedes* mosquitos (1). It is caused by the yellow fever virus, a reemerging virus that is endemic in several sub-Saharan and South American countries. Due to its severity and the high risk of widespread outbreaks, most countries where it is endemic implement yellow fever surveillance. In this surveillance, yellow fever is often associated with clinical cases of acute febrile jaundice (2). However, this clinical syndrome is common to several endemic diseases, particularly viral hepatotropic infections (3).

There are currently five main unrelated hepatotropic viruses, referred to as the hepatitis A virus (HAV), the hepatitis B virus (HBV), the hepatitis C virus (HCV), the hepatitis D virus (HDV), and the hepatitis E virus (HEV) (4). HAV and HEV are waterborne viruses that usually cause acute hepatitis without progressing to chronic liver diseases (5, 6). According to the Global Burden of Disease study, 101.7 million cases of HAV and 28.4 million cases of HEV infection occurred worldwide in 2013 (7). Both viruses cause sporadic cases and outbreaks, most of which occur in under-resourced settings (8, 9). In Africa, HEV outbreaks are reported nearly every year, and some outbreaks involved more than 10,000 cases (5, 10). HAV is highly endemic in sub-Saharan Africa where most children have serological evidence of a prior infection by their fifth birthday (11). Given that long-term immunity is conferred after HAV infection, epidemics are uncommon in such areas where this virus is endemic (8). However, the lack of systematic monitoring suggests that HAV and HEV prevalences are underestimated in Africa (5, 12).

HBV, HCV, and HDV are transmitted by parenteral, sexual, or mother-to-child routes. They usually evolve into chronic hepatitis, liver cirrhosis, and hepatocellular carcinoma resulting in high mortality and morbidity (13–15). Moreover, superinfection of HBV patients with HDV, a small defective virus that requires the HBV envelope for its replication, frequently accelerates the progression of HBV disease to liver cirrhosis, considerably increasing the burden of HBV-related chronic liver diseases (14, 16). Worldwide, more than 350 million people are chronically infected with HBV, 150 million with HCV, and 15 million with HDV (13–15). Sub-Saharan Africa has some of the highest hepatitis prevalence rates in the world, ranging from 3 to 20% for the HBV surface antigen (17) and from 1 to 7% for HCV (18) in the general population. Similarly, HDV prevalence estimates have revealed large disparities among countries in which carriers of the HBV surface antigen (HBsAg) reside, ranging from 0% in Mozambique to 70.6% in an urban area in Gabon (19, 20).

In the Democratic Republic of Congo (DRC), the Ministry of Health began yellow fever surveillance in 2003 as part of an integrated disease surveillance program. Case detection is based on a standard case definition, and reporting occurs at the local health zone level.

Blood samples from suspected cases of yellow fever are collected and forwarded to the national reference laboratory in Kinshasa, which is responsible for the laboratory diagnosis of all samples from the integrated disease surveillance in the DRC. Acute febrile jaundice cases have frequently been reported from the yellow fever surveillance program. Of these suspected cases of yellow fever, very few (<5%) are actually confirmed by laboratory testing (i.e., by enzyme-linked immunosorbent assay [ELISA]). Although acute febrile jaundice strongly suggests infection with hepatitis viruses in medical practice, patients who test negative for yellow fever are never screened for these viruses. Furthermore, viral hepatitis prevalence data in the DRC are either absent (HDV), scarce (HEV) (21), old (HAV) (22), or limited to asymptomatic patients in urban areas (HBV and HCV) (23–25). Little is known about the prevalence and genetic diversity of these viruses in symptomatic patients. The aim of this study was to screen for HAV, HBV, HCV, HDV, and HEV in patients included in the yellow fever surveillance program in the DRC and who tested negative for the yellow fever virus by ELISA and PCR.

## RESULTS

### Serological and virological findings.

A total of 43.7% (218/498) of serum samples were infected with at least one of the five investigated hepatitis viruses; this value is nearly identical to the prevalence of infection among samples successfully tested for all four independent viruses (45.9% [185/403]). The most prevalent virus was HBV with 22.3% (105/470) of HBsAg-positive individuals and 11.0% (49/444) with anti-hepatitis B core protein (HBc) IgM antibodies, of which 22.4% (11/49) of patients were HBsAg-negative (Table 1). HBV DNA was quantified in 83 serological HBV-positive samples. The majority of patients positive for either HBsAg or anti-HBc IgM (95.0% [*n* = 83]) had detectable levels of HBV DNA (>100 IU/ml). The median value was 4.19 × 10^5^ IU/ml (range, 769 to 9.82 ×10^9^ IU/ml). Most HBc IgM-positive samples (82.3%) carried viral loads higher than 10^4^ IU/ml. Three of 11 HBsAg-negative and HBc IgM-positive samples tested had viral DNA levels higher than 10^3^ IU/ml. Unfortunately, there was not enough sera to analyze the eight remaining samples. Twenty-two HBsAg-positive patients (26.1%) tested positive for HDV, of which 15 (68.2%) were positive for anti-HDV antibodies and 7 (31.8%) were positive for the HDV antigen, suggesting primary HDV infection (26). HBV viremia was higher in HBV mono-infected patients (mean, 2.9 × 10^8^ IU/ml) than in HBV-HDV coinfected patients (mean, 9.6 × 10^7^ IU/ml), but the difference was not significant (Wilcoxon test, *W* = 412.5, *P* = 0.729). A total of 13 samples were tested for HDV RNA, 12 (92.3%) had detectable levels of RNA, and viral load was quantified for 10 samples at a median value of 1.4 × 10^6^ IU/ml (range, 3.1 × 10^2^ to 2.9 × 10^8^ IU/ml). Among samples with detectable RNA, three were positive for HBc IgM.

**TABLE 1:** Prevalence and demographic characteristics of viral hepatitis markers among patients with jaundice who tested negative for yellow fever in the DRC^*a*^

Characteristic	HBV surface Ag^*b*^ (*n* = 470)	Anti-HBc^*c*^ IgM (*n* = 444)	HDV Ag + total Ab^*d*^ (*n* = 84)	HCV Ag + total Ab (*n* = 379)	HAV IgM (*n* = 432)	HEV IgM (*n* = 365)
Overall prevalence (% [95% CI])	22.3 (18.5–26.0)	11.0 (8.1–13.9)	26.1 (16.6–35.5)	2.3 (0.8–3.8)	16.7 (13.2–20.2)	10.4 (7.3–13.5)
Sex (female/male, 0.8:1)						
Male	65/257 (25.2)	23/243 (9.4)	13/49 (26.5)	5/201 (2.4)	39/233 (16.7)	25/195 (12.8)
Female	40/213 (18.7)	26/201 (12.9)	9/35 (25.7)	4/178 (2.2)	33/199 (16.5)	13/170 (7.6)
Age (mean, 22.4 yr; range, 4 mo to 77 yr)						
0–5 yr	13/118 (11)	7/107 (6.5)	2/10 (20)	2/90 (2.2)	46/108 (42.5)	0/87 (0)
6–15 yr	14/78 (17.9)	6/76 (7.8)	2/12 (16.6)	0/64 (0)	21/74 (28.3)	2/67 (2.9)
16–40 yr	59/184 (32)	29/176 (16.4)	12/45 (26.6)	3/146 (2)	4/166 (2.4)	22/142 (15.4)
>40 yr	19/90 (21.1)	7/85 (8.2)	6/17 (35.2)	4/79 (5)	1/84 (1.1)	14/69 (20.2)
Province of origin						
Bandundu	9/34 (26.4)	8/34 (23.5)	0/8 (0)	0/22 (0)	4/32 (12.5)	0/29 (0)
Bas-Congo	4/14 (28.5)	2/13 (15.3)	0/3 (0)	0/10 (0)	5/13 (38.4)	0/11 (0)
Equateur	31/121 (25.6)	11/112 (9.8)	9/27 (33.3)	1/99 (1)	12/116 (10.3)	28/98 (28.5)
Kasai-Occidental	12/48 (25)	7/47 (14.8)	0/13 (0)	1/31 (3.2)	4/45 (8.8)	8/37 (21.6)
Kasai-Oriental	9/52 (17.3)	6/54 (11.1)	1/8 (12.5)	1/40 (2.5)	8/54 (14.8)	1/44 (2.2)
Katanga	0/6 (0)	0/6 (0)	0/0	0/6 (0)	0/6 (0)	0/3 (0)
Kinshasa	3/21 (14.2)	2/21 (9.5)	1/2 (50)	0/14 (0)	4/21 (19)	0/15 (0)
Maniema	0/11 (0)	0/11 (0)	0/0	1/10 (10)	2/11 (18.1)	0/9 (0)
Nord-Kivu	0/11 (0)	0/11 (0)	0/0	0/10 (0)	1/11 (9)	0/11 (0)
Orientale	37/152 (24.3)	13/135 (9.6)	11/23 (47.8)	5/137 (3.6)	32/123 (26)	1/108 (0.9)

a Data are number of samples testing positive/total number of antibodies tested (%) unless stated otherwise.

b Ag, antigen.

c Hbc, hepatitis B core protein.

d Ab, antibody.

Presumed acute infection with HAV and HEV, as defined by the presence of anti-HAV IgM and anti-HEV IgM, which predominate the primary immune response, was revealed in 72 (16.7%) and 38 (10.4%) patients, respectively. A total of 9 (2.3%) patients showed evidence of serological HCV infection. We also observed 17 (3.4%) cases of concomitant infections among independent viruses (Table 2). Of 72 patients infected with HAV, 7 (9.7%) were coinfected with HBV, and among HEV-infected patients, 9 (23.6%) were coinfected with HBV. One patient was coinfected with HBV and HCV, and another showed a triple infection with HBV, HDV, and HEV.

**TABLE 2:** Hepatitis virus identities in coinfected patients^*a*^

Patient ID	Date collected	HBV	HDV	HCV	HAV	HEV
218	24 October 2011	1	NA	0	1	0
331	1 September 2006	1	1	NA	0	1
443	5 July 2009	1	0	NA	1	0
118	3 August 2010	1	0	0	1	0
335	18 September 2006	1	0	NA	0	1
407	28 November 2008	1	0	NA	0	1
316	31 August 2006	1	0	NA	0	1
301	31 August 2006	1	0	NA	0	1
326	31 August 2006	1	0	NA	0	1
337	26 December 2006	1	0	NA	1	NA
2	8 January 2010	1	0	1	0	0
420	5 January 2009	1	0	NA	0	1
378	8 May 2008	1	0	NA	1	0
372	3 May 2008	1	0	NA	0	1
160	13 August 2010	1	0	0	1	0
144	10 August 2010	1	NA	0	1	0
455	20 September 2009	1	0	NA	0	1

a ID, identification; NA, not analyzed; 1, positive; 0, negative.

### Sociodemographic characteristics of positive viral hepatitis samples.

### Demographic results.

Analysis of the prevalences of HBV and HEV among age groups revealed statistically significant differences in the patterns of occurrence (HBV: χ^*2*^ = 21.3, df = 3, *P* < 0.001; HEV: χ^*2*^ = 25.2, df = 3, *P* < 0.001) (Table 1). Although HAV infection occurred mostly in children 15 years old or younger (χ^*2*^ = 89.4, df = 1, *P* < 0.001), HEV infection was observed widely in adults older than 15 years (χ^*2*^ = 22.1, df = 1, *P* < 0.001). HBV and HDV coinfections were most frequently found in adults aged 16 years or older (18 of 22 cases), but this pattern was not statistically significant (*P* = 0.48), because HBV was also common among adults (χ^*2*^ = 13.4, df = 1, *P* < 0.001). There were no differences in HCV, HDV, or HAV prevalences between men and women. Likewise, the sex difference observed in HBV and HEV prevalence was not statistically significant (*P* > 0.05).

### Geographical distribution.

**FIG 1:**
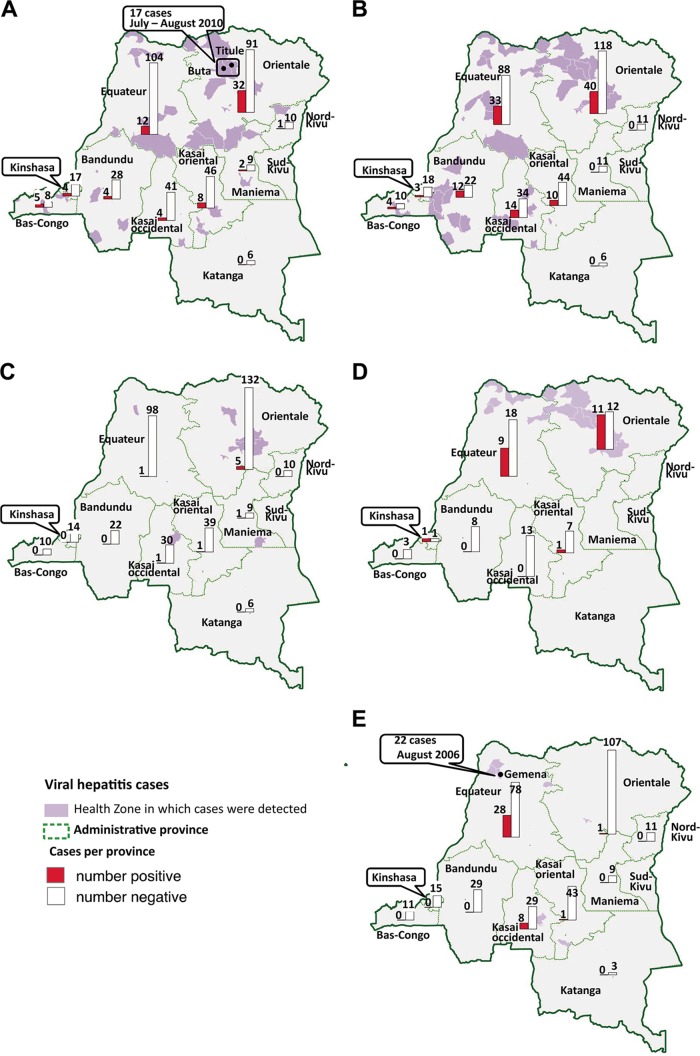
Maps of hepatitis virus distribution in provinces of the Democratic Republic of Congo. The numbers of positive (red) and negative (white) samples tested in each province are depicted by the bar charts, with the exact numbers given above the bars. The specific health zones in which positive samples were detected are shaded in purple. Green lines delimit administrative provinces. (A) Distribution of hepatitis A virus IgM antibodies in suspected cases of yellow fever by province. Buta and Titule are two cities in which cases of HAV were detected during the yellow fever epidemic in 2010. (B) Distribution of hepatitis B virus in suspected cases of yellow fever by province. (C) Distribution of hepatitis C virus in suspected cases of yellow fever by province. (D) Distribution of hepatitis D virus in suspected cases of yellow fever by province. (E) Distribution of hepatitis E virus IgM antibodies in suspected cases of yellow fever by province. The city of Gemena, where a high number of cases of HEV were recorded in August 2006, is identified on the map.

Hepatitis viruses were detected in patients from almost all sampled provinces of the DRC except in Katanga (Fig. 1). Most of the cases recorded in this study originated from the two northern-most provinces of Equateur and Orientale, where 66 (30.3%) and 75 cases (34.4%), respectively were infected with at least one hepatitis virus (see Fig. S1 in the supplementary material). There were nonsignificant trends for geographic structure in HBV prevalence among provinces (χ^*2*^ = 14.57, df = 9, *P* = 0.103; Table 1), but we observed a significantly higher HAV prevalence in Orientale (χ^*2*^ = 9.9, df = 1, *P* = 0.0016) and higher HEV prevalence in Equateur and Kasai-Occidental (together, χ^*2*^ = 58.0, df = 1, *P* < 0.001) than the rest of the country, especially in the town of Gemena (Equateur), where 22 of 28 patients were HEV positive. Unlike HBV distribution, HDV-positive cases were found almost exclusively in the two northern-most provinces of the country (Fig. 1; Equateur and Orientale versus the rest of the country, χ^*2*^ = 10.49, df = 1, *P* = 0.0012).

### Prevalence trends over time.

**FIG 2:**
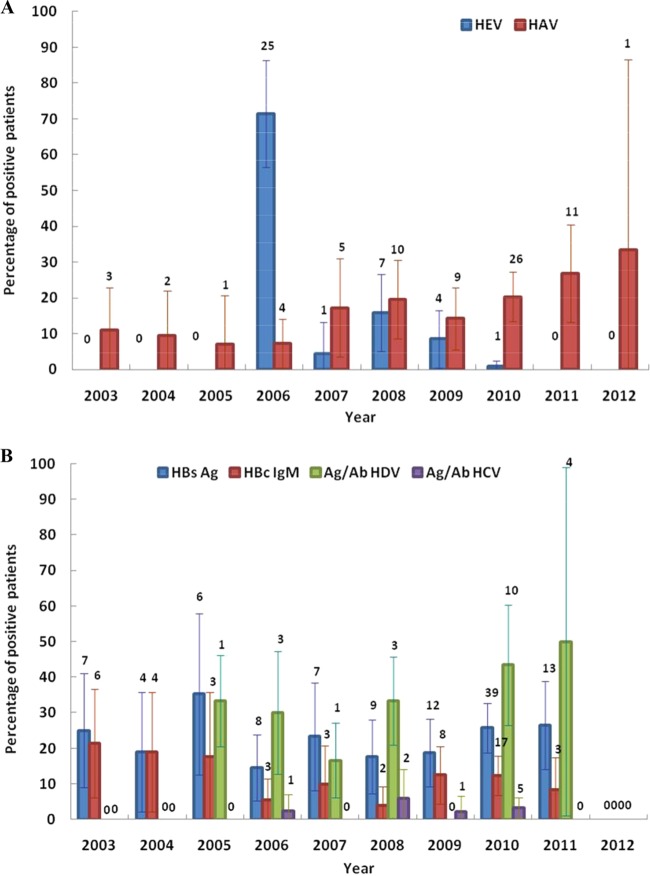
(A) Changes in hepatitis A virus and hepatitis E virus prevalence over time (from January 2003 to January 2012). Red bars show the prevalence of patients with hepatitis A virus IgM antibodies and blue bars show the prevalence of patients with hepatitis E virus IgM antibodies. Numbers above the error bars show the actual numbers of samples that tested positive. (B) Changes in hepatitis B virus, hepatitis C virus, and hepatitis D virus prevalences over time (from January 2003 to January 2012). Shown are the prevalence rates of HBsAg (blue), anti-HBc IgM (red), hepatitis D virus antigen and total antibodies (green), and hepatitis C virus antigen and total antibodies (purple). Numbers above the error bars show the actual numbers of samples that tested positive.

HAV cases were always detected without significant geographic concentrations during the study period. Among patients enrolled in July and August 2010, 21.4% and 32.1%, respectively, were positive for HAV and all came from the towns of Titule and Buta (Orientale). The overall HEV frequency was very high in 2006 compared with during the rest of the study period (Fig. 2). All 25 HEV cases detected in 2006 were from Equateur. A total of 22 HEV-positive patients, representing 78.5% of patients enrolled in August 2006, came from the town of Gemena. HBV, HDV, and HCV prevalences remained relatively stable over time (Fig. 2).

### HBV and HDV genotyping and phylogenetic analyses.

**FIG 3:**
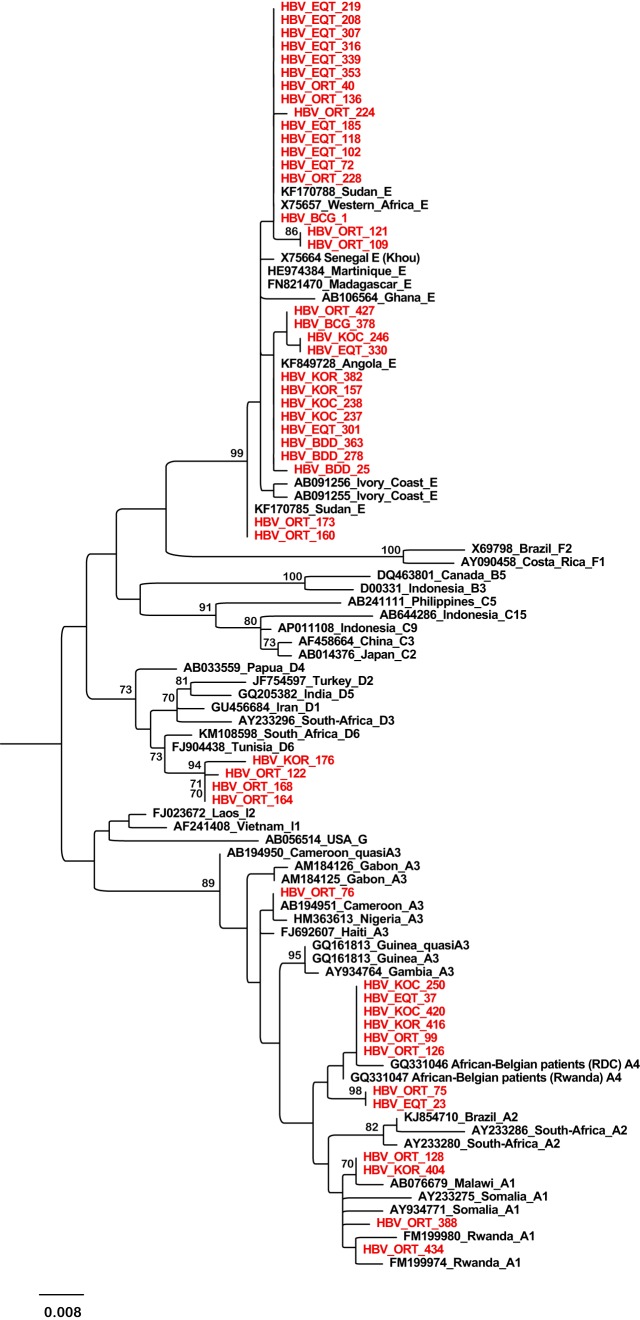
Phylogenetic tree of a 497-bp fragment of the HBV S gene obtained from 48 patients with acute febrile jaundice and from 48 reference HBV sequences. Only bootstrap values ≥70 are shown (500 replicates). Sequences from this work are shown in red and reference sequences are labeled with their accession numbers and country of origin. The letters A, B, C, D, E, F, G, H, and I indicate the HBV genotype and the numbers indicate the subgenotypes.

The HBV S fragment (nucleotide [nt] 278 to 775) was successfully amplified and sequenced from 48 HBV DNA-positive samples. As shown in Fig. 3, we detected the HBV-E (*n* = 31), HBV-A (*n* = 13), and HBV-D (*n* = 4) genotypes. The identified HBV-E strains were from six different DRC provinces, including northern provinces (Equateur and Orientale) and southwestern provinces (Bas-Congo, Bandundu, Kasai-Occidental, and Kasai-Oriental). These HBV-E strains all clustered together regardless of their province of origin. Within genotype D, all four sequences formed a distinct subcluster, supported by a high bootstrap value of 94 and clustered with the D6 subgenotype reference strains. Among the 13 genotype A isolates, 4 clustered with subgenotype A1, 8 with subgenotype A4, and the last one clustered within subgenotype quasi-A3. In general, sequences from the DRC were closely related to the corresponding sequences from neighboring countries or other African countries.

**FIG 4:**
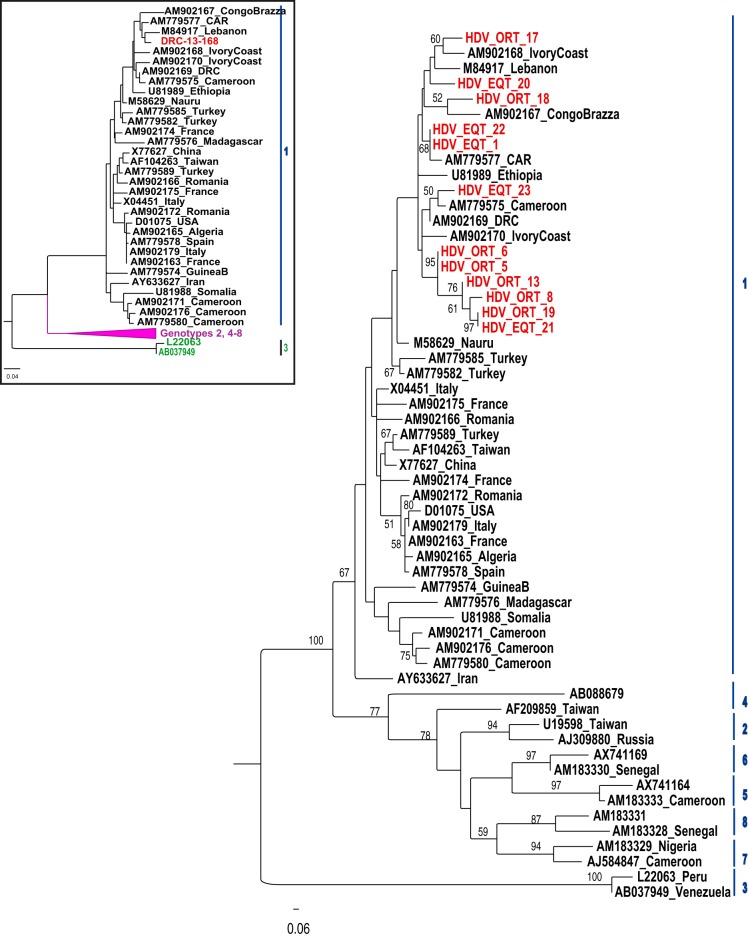
Phylogenetic tree of a 329-bp fragment of the HDV R0 region using the neighbor-joining algorithm. For phylogenetic analysis, 12 HDV sequences from the DRC (shown in red) were aligned with 45 available full-length genomic sequences using MEGA7 software. Numbers on the nodes of the tree give the bootstrap values of the nodes (500 replicates). The inset shows a rooted phylogenetic tree of the complete genome determined from one HDV sequence with the 45 available full-length sequences using the neighbor-joining method. The complete genome analysis confirmed the analysis based on the R0 gene.

For sequencing and phylogenetic characterization, the HDV R0 region was successfully amplified from 12 samples from the two northern provinces. As shown in Fig. 4, all sequences belonged to HDV clade 1 and clustered together with African reference isolates from the “African-Middle East” branch of the HDV 1 tree as described previously (27). We found two identical DRC sequences (HDV_ORT_6 and HDV_ORT_5), which were characterized from patients from the same city. We also isolated two other very similar sequences, HDV_EQT_21 from Equateur and HDV_ORT_19 from Orientale, indicating intensive and ongoing HDV transmission throughout the northern part of the country. The complete HDV genome sequence was obtained for one of the strains and the phylogenetic analysis confirmed its African HDV-1 lineage (Fig. 4).

## DISCUSSION

Nearly 10 years of monitoring yellow fever has shown that the large majority of samples collected from patients with acute febrile jaundice in the DRC are not diagnosed with yellow fever. However, this disease shares the same symptoms with several other diseases, such as viral hepatitis, which are not routinely investigated in undiagnosed patients. The aim of the present study was to screen for HAV, HBV, HCV, HDV, and HEV infections in patients with acute febrile jaundice detected through the yellow fever surveillance program in the DRC.

In total, 218 of the 498 patients (43.7%) of the yellow fever-negative cohort (IgM and PCR negative) were positive for at least one hepatitis virus, with most of the cases originating from the two northern-most provinces of Equateur (30.3%) and Orientale (34.4%). HBV infection was predominant, with virtually half of the infected patients being in the acute phase, whereas only a few cases of HCV infection were identified. We also found a high proportion of HDV infection in HBsAg-reactive patients, almost exclusively from the northern provinces of the country. Acute HAV and HEV infections were detected in 16.7% and 10.4% of patients tested, respectively.

Hepatitis viruses were mostly associated with acute febrile jaundice in the yellow fever-negative cohort, especially in the two northern provinces of the DRC. The potentially explanatory epidemiological data and sociocultural factors were not documented, because—in accordance with WHO recommendations—yellow fever suspicions are based on the standard case definition. Only minimal information, such as age, sex, date of collection, and the collection site, are recorded. Nevertheless, the majority of samples included in the yellow fever surveillance were collected in the two northern provinces, mainly during a laboratory-confirmed outbreak of yellow fever that hit Titule and Buta health zones in 2010 (Orientale) from June to August. Sporadic yellow fever cases and outbreaks are frequently reported in these two provinces, heightening awareness among the population and health care staff in this area. Therefore, yellow fever diagnosis is readily suspected at the slightest warning sign, resulting in high rate of reported suspected cases of yellow fever compared with in the other provinces that do not experience yellow fever outbreaks. In addition, yellow fever surveillance uses a broad case definition that further increases the probability of encompassing several diseases with similar clinical symptoms, such as acute viral hepatitis, especially because differential laboratory diagnosis is not often available, due either to the lack of equipped laboratories or to the prohibitive cost of analyses.

HBV infection still threatens Congolese public health despite all efforts deployed in the DRC. In our cohort, HBsAg prevalence (22.3%) was higher than that previously reported in asymptomatic blood donors in Kinshasa town and Lubumbashi (more than 9%) (28, 29), Kisangani (5.4%) (30), and Bukavu (4.8%) (25). Although HBV infection often becomes chronic, we revealed a high rate of acute HBV infection based on anti-HBc IgM reactivity (11.0%) along with high HBV DNA levels (82.3%). This result suggests that the clinical symptoms of acute febrile jaundice observed in these patients, initially suspected of yellow fever, may be associated with HBV infection. The prevalence of HBsAg and anti-HBc IgM increased with age and was significantly high among adults older than 15 years (*P* < 0.001). Therefore, HBV horizontal transmission appears to be high, with sexual transmission and high-risk behavior potentially playing a major role in the DRC (13). Horizontal transmission appears to predominate in several sub-Saharan African countries, such as Gabon (13, 31). Government policy has focused primarily on screening blood donors. The introduction of an HBV vaccine in the routine immunization schedule dates only from 2008. It is a three-dose vaccine, the first administered 45 days after birth. However, according to WHO estimations, vaccine coverage is low (70% in 2012), and infants under 45 days of age are at risk. In light of our results, extensive health education combined with vaccination and blood screening measures must be implemented to limit disease transmission in the current unimmunized population, i.e., the vast majority of Congolese people. A study conducted in 2004 in seven sub-Saharan African countries reported the presence of three genotypes (E, A, and D) in six samples collected from the DRC (32). Here, we confirmed these data and demonstrated that genotype E was the most prevalent, being detected in 65.9% genotyped samples collected from 6 of the 10 sampled provinces. This genetic diversity is consistent with the geography of the DRC, located at the crossroads of regions where HBV genotypes E, A, and D have been reported, namely, HBV/E in West and Central Africa, HBV/A in southern, East, and Central Africa, and HBV/D in North Africa (33). Neighboring countries such as Angola, the Central African Republic (CAR), Uganda, and Sudan have also reported the presence of these three genotypes (33–36). Based on phylogenetic analysis of the S gene fragment, HBV/A sequences clustered with the A1, quasi-A3, and A4 subgenotype reference groups, and HBV/D sequences formed a distinct cluster close to the D6 reference subgenotype. However, this position was not supported by significant bootstrap values (less than 70%). Further studies with full genome characterization are needed to accurately identify the relationships among subgenotypes, especially because recombinant strains have been reported in neighboring countries, such as Sudan, the CAR, Kenya, Gabon, and Cameroon (35, 37–40).

In this study, we described the prevalence, genotype, and geographic distribution of HDV infection in the DRC for the first time. We found 26.1% HDV seropositivity in HBsAg-reactive patients of the negative yellow fever cohort. High rates of HDV antibodies have been reported also in patients with hepatocellular carcinoma in the CAR (53%) (41) and in rural Uganda (30.6%) (42). Although HBV and HDV are strongly related and have the same transmission routes, the prevalence pattern of HDV did not geographically match that of HBV. Almost all HDV-positive patients (20/22) were from the northern DRC and phylogenetic analysis of HDV R0 sequences, confirmed by the analysis of the complete genome, showed that DRC sequences belonged to genotype 1 and were similar to the neighboring CAR strain. The northern DRC seems to be a hotspot for HDV infection, being close to the CAR and Uganda where HDV is endemic. Although none of the factors associated with high transmission were identified in this study, it is possible that sociocultural factors contribute to this high rate of transmission, owing to the cultural and linguistic communities of ethnic groups living in the northern DRC. This finding is in agreement with results from previous studies that have shown that HDV prevalence varies greatly among and within countries and, even in regions of high endemicity, the prevalence of HDV is not necessarily proportional to that of HBV (43). Large geographical differences in HDV prevalence have also been observed in sub-Saharan Africa. Prevalence rates of 15.6% in Gabon (44), 17.6% in Cameroon (45), and 31.0% in northern Kenya (46) have been reported, whereas no patients tested positive for HDV antibodies in Burundi (personal data), Tanzania, or Mozambique (19). However, as most patients in this study were from the northern part of the country, more extensive epidemiological studies are needed to verify this geographic distribution and assess the extent and the characteristics of HDV infection in the DRC.

HCV seroprevalence (2.3%) was similar to the rates reported in asymptomatic blood donors and pregnant women from sub-Saharan African countries (47). A systematic review of HCV published studies in the DRC has reported large differences in anti-HCV prevalence between blood donors (0.2 to 4.1%) and some high-risk populations such as military personnel (13.7%). Nevertheless, the overall prevalence of 2.9% estimated in the reviewed studies was similar to our finding (48). Since most of these studies were performed in asymptomatic people, our finding may be explained by the fact that acute HCV infection is asymptomatic in the majority of cases (49, 50). Screening for HAV and HEV is rare, even in areas where they are endemic, because the infection is generally asymptomatic and self-limited. In this study, HAV was the most important cause of acute infection identified in acute febrile jaundice patients detected through the yellow fever surveillance program. The virus was detected throughout the country, mainly in children 15 years of age and under. To our knowledge, only one study has reported a 99% prevalence of anti-HAV antibodies in a rural population in the northern DRC in 1985 (22). These results show that HAV is likely endemic to the DRC. Here, we also reported a high prevalence of HEV infection. This rate was likely due to the increase in HEV infection in August 2006 in the town of Gemena observed in our study. With the exception of this period of increase, the rate of HEV infection remained at a background level. The cause of this sudden increase is unknown, but it appears that a hepatitis E outbreak went unnoticed in this city. In a context in which access to diagnostic testing is relatively limited, such epidemiological findings are of general interest and further emphasize the need for including routine serological tests for HAV and HEV for differential diagnosis of viral hepatitis in the DRC, especially during outbreaks. Early diagnosis of these infections during outbreaks enables timely intervention to control transmission.

In conclusion, we showed that viral hepatitis types A, B, C, D, and E are very important causes of acute febrile jaundice in patients included in the yellow fever surveillance program in the DRC. This finding highlights the necessity to also screen for hepatitis viruses in the yellow fever surveillance program. To reduce the spread of these serious diseases, an effective viral hepatitis control program needs to be implemented. This program should not only include prevention strategies, but also should strive to enhance diagnostic capabilities and organize the follow-up of patients. Therefore, further epidemiological studies are required to determine the specific areas where HBV/HDV is highly endemic in particular and to identify the risk factors behind transmission.

## MATERIALS AND METHODS

### Sample characterization.

Samples were collected in the yellow fever surveillance program from suspected cases of yellow fever, defined as patients with an acute onset of fever followed by jaundice within 2 weeks of the first symptoms. Of the 652 samples collected in 10 of the 11 DRC provinces from January 2003 to January 2012, 498 samples that had a volume sufficient for at least biological exploration were included in this study. The majority of patients were from the two northern provinces, including 175 (35.1%) patients from the Orientale province and 123 (24.7%) patients from the Equateur province. In the other provinces, the number of patients ranged from 6 to 54. The male-to-female ratio was virtually 1:1 with 269 (54.0%) male patients and 229 (46.0%) female patients. Patient ages ranged from 4 months to 77 years (mean, 22.4 years). The recruitment rates varied over the study years, being very high in 2010 (35.1%), followed by 2009 (12.9%), 2008 (10.2%), and 2006 (11.0%). For the remaining years, the recruitment rate was lower than 10%. In 2010, most of the patients were enrolled in July (48.5%) and in August (20%), mostly from two neighboring cities, Titule and Buta (Orientale), during a laboratory-confirmed outbreak of yellow fever. In 2006, 63.6% of patients were enrolled in the town of Gemena (Equateur) in August. During the rest of the study period, there was no obvious concentration of cases.

### Preliminary laboratory analysis.

Each case was initially screened for malaria using the thick blood smear method. Then, samples that tested negative for malaria and those collected from patients whose illness failed to respond to antimalarial drugs were transferred to the Institut National de Recherche Biomédicale (INRB), the national public health laboratory, to screen for yellow fever antibodies using the IgM-capture ELISA. Positive samples were sent to the regional reference laboratory for yellow fever at the Institut Pasteur in Dakar, Senegal for confirmation with more specific tests (i.e., plaque-reduction neutralization test). The remaining positive sera were used as internal positive controls, and they were therefore not included in this study. Negative sera were stored at −20°C until their transfer to the Centre International de Recherches Médicales de Franceville (CIRMF) in Gabon where they were screened for hepatitis viruses after confirming the negative yellow fever status using semiquantitative TaqMan PCR as described elsewhere (51).

### Ethical considerations.

The study was conducted with the authorization of the DRC Ministry of Health and the WHO to supplement yellow fever surveillance data in the DRC. Data collected from the INRB laboratory database of yellow fever surveillance remained confidential and, at the end of the study, results were made available to the Ministry of Health.

### Serological tests.

We first determined the presence of three chronic hepatitis viruses, namely, HBV, HDV, and HCV. HBsAg was first assayed using the VIDAS HBsAg ultra (bioMérieux, Lyon, France) commercial enzyme-linked fluorescence assay (ELFA) and the presence of anti-HBc IgM was determined using the Monolisa HBc IgM plus kit (Bio-Rad, Marnes-la-Coquette, France). HDV antibodies and antigen status were determined using the ETI-AB-DELTAK-2 (P2808; DiaSorin, Saluggia, Italy) and ETI-DELTAK-2 (P002097; DiaSorin) kits, respectively, in HBsAg-positive patients. Antigen and total antibodies against HCV were tested using the Monolisa HCV Ag-Ab ultra kit (Bio-Rad). We then tested for the presence of two acute hepatitis viruses, HAV and HEV. HAV IgM antibodies were screened using the Monolisa HAV IgM plus kit (Bio-Rad), and HEV IgM antibodies were tested using the HEV-IgM ELISA kit (Wantai, Beijing, China). All analyses were performed according to the manufacturers' instructions.

### RNA and DNA isolation.

Due to insufficient amounts of sera, RNA and DNA extractions were performed only on HBV-infected patients. Viral DNA and RNA were extracted from 200 μl of serum and eluted in 120 μl of elution buffer using the EZ1 advanced XL BioRobot (Qiagen, CA, USA) with the EZ1 virus minikit v2.0 (Qiagen) according to the manufacturer's instructions.

### HBV and HDV quantification.

To determine the HBV viral load using real-time PCR, we used forward primer primHBV1 (5′-GTGTCTGCGGCGTTTTATCA-3′) and reverse primer primHBV2 (5′-AGGCATAGCAGCAGGATGAA-3′) with a probe (5′-FAM-TGCGGCGTTTTATCAT-MGB-3-′). This primer/probe set was designed during the Agence Nationale de Recherches sur le Sida et les hépatites virales (ANRS 12187) project; it targets a conserved region in the HBV S gene (nt positions 379 to 426). Amplification was performed as described previously (52).

To determine the HDV viral load, we used the protocol described in 2005 by Le Gal et al. (53). Briefly, cDNAs were synthesized with random hexamers and purified with Montage PCR centrifugal filter devices (Millipore, Molsheim, France). Real-time PCRs were then performed in an ABI7500 thermocycling system (Applied Biosystems, Courtaboeuf, France) using the TaqMan universal PCR master mix (Applied Biosystems) and specific primers and probes designed to target the two ribozymes.

### Phylogenetic analysis.

The HBV genotype was determined by phylogenetic analysis of a 497-bp fragment of the HBV S gene. The fragment was obtained by PCR and nested PCR using Taq DNA Core kit 25 (MP Biomedicals, France) and primers PS1F (5′-TCACAATACCGCAGAGTCT-3′) and PS1R (5′-AACAGCGGTATAAAGGGACT-3′) for the first round and PS2F (5′-GTGGTGGACTTCTCTCAATTTTC-3′) and PS2R (5′-CGGTATAAAGGGACTCACGAT-3′) for the second round of PCR. Amplifications were performed using the same conditions as described elsewhere (54). PCR products were sequenced bidirectionally using BigDye terminator technology. The sequences obtained were compared to 48 previously published sequences representing each HBV genotype, from A to H.

The HDV genotype was determined by phylogenetic analysis of the R0 region of the genome as described previously (55). Amplification of this region used primers 1289as (5′-GAAGGAAGGCCCTCGAGAACAAGA-3′) and 920s (5′-CATGCCGACCCGAAGAGGAAAG-3′), covering the 19 specific amino acids of the large delta protein that discriminates the eight known HDV genotypes. PCR products were sequenced bidirectionally using BigDye terminator technology and were compared to 45 reference HDV sequences, including known genotypes 1 to 8. The complete sequence of the genome was fully characterized by amplification and sequencing of four overlapping regions (R0, nt 889 to 1,289; R1, nt 320 to 1,289; R2, nt 889 to 420; and R3, nt 200 to 560) as described elsewhere (56). Phylogenetic analysis was performed on the obtained sequences as well as the 45 available full-length genomic sequences representing the eight HDV genotypes.

Raw HDV and HBV sequences were edited and manually corrected using Genious R7. Maximum-likelihood trees and genetic distances were calculated using MEGA7 software with the Kimura 2-parameter/gamma model for HBV and Hasegawa-Kishino-Yano for HDV R0, and 500 bootstrap replicates (57). For the complete genome, we used the neighbor-joining (NJ) method and the Kimura 2-parameter model.

### Statistical analysis.

Prevalences are described with 95% confidence intervals (CIs), and chi-square test was used to compare the proportions of infection according to age, sex, and geographical region. For quantitative variables, the medians were calculated and the Wilcoxon test was used to compare HBV viral loads in HDV-positive and HDV-negative patients. Statistical significance was assessed at a *P* value of <0.05. Analyses were performed using IBM SPSS statistics software, version 20.

### Accession number(s).

The new HBV strains and HDV sequences obtained in this study are available under European Nucleotide Archive (ENA) accession numbers LT714643 to LT714690 and LT703299 to LT703311, respectively.

## Supplementary Material

Supplemental material

## References

[1] Yaro S, Ouoba AR, Zango A, Rouamba J, Drabo A, Ouangraoua S, Samandoulougou-Kirakoya F, Macq J, Robert A, Ouedraogo JB 2013 Management problems of trans-frontier yellow fever cases in Burkina Faso 2010. Adv Infect Dis 3:84–88. doi:10.4236/aid.2013.32013.

[2] World Health Organization. 1998 District guidelines for yellow fever surveillance. World Health Organization, Geneva, Switzerland.

[3] Adungo F, Yu F, Kamau D, Inoue S, Hayasaka D, Posadas-Herrera G, Sang R, Mwau M, Morita K 2016 Development and characterization of monoclonal antibodies to yellow fever virus and application in antigen detection and IgM capture enzyme-linked immunosorbent assay. Clin Vaccine Immunol 23:689–697. doi:10.1128/CVI.00209-16. 27307452 PMC4979174

[4] Dudley T 2009 Viral hepatitis, p 83–92. *In* Sargent S (ed), Liver diseases: an essential guide for nurses and health care professionals, 2nd ed Blackwell Publishing, Ltd, Oxford, United Kingdom.

[5] Kim JH, Nelson KE, Panzner U, Kasture Y, Labrique AB, Wierzba TF 2014 A systematic review of the epidemiology of hepatitis E virus in Africa. BMC Infect Dis 14:308. doi:10.1186/1471-2334-14-308. 24902967 PMC4055251

[6] Joon A, Rao P, Shenoy SM, Baliga S 2015 Prevalence of hepatitis A virus (HAV) and hepatitis E virus (HEV) in the patients presenting with acute viral hepatitis. Indian J Med Microbiol 33 Suppl:102–105. 25657123 10.4103/0255-0857.150908

[7] Global Burden of Disease Study 2013 Collaborators. 2015 Global, regional, and national incidence, prevalence, and years lived with disability for 301 acute and chronic diseases and injuries in 188 countries, 1990–2013: a systematic analysis for the Global Burden of Disease Study 2013. Lancet 386:743–800. doi:10.1016/S0140-6736(15)60692-4. 26063472 PMC4561509

[8] Jacobsen KH, Koopman JS 2005 The effects of socioeconomic development on worldwide hepatitis A virus seroprevalence patterns. Int J Epidemiol 34:600–609. doi:10.1093/ije/dyi062. 15831565

[9] Kmush B, Wierzba T, Krain L, Nelson K, Labrique AB 2013 Epidemiology of hepatitis E in low- and middle-income countries of Asia and Africa. Semin Liver Dis 33:15–29. doi:10.1055/s-0033-1338111. 23564386

[10] Spearman CW, Sonderup MW 2015 Health disparities in liver disease in sub-Saharan Africa. Liver Int 35:2063–2071. doi:10.1111/liv.12884. 26053588

[11] Jacobsen KH 2014 Hepatitis A virus in West Africa: is an epidemiological transition beginning? Niger Med J 55:279–284. doi:10.4103/0300-1652.137185. 25114360 PMC4124538

[12] Gerbi GB, Williams R, Bakamutumaho B, Liu S, Downing R, Drobeniuc J, Kamili S, Xu F, Holmberg SD, Teshale EH 2015 Hepatitis E as a cause of acute jaundice syndrome in northern Uganda, 2010–2012. Am J Trop Med Hyg 92:411–414. doi:10.4269/ajtmh.14-0196. 25448237 PMC4347349

[13] Kramvis A, Kew MC 2007 Epidemiology of hepatitis B virus in Africa, its genotypes and clinical associations of genotypes. Hepatol Res 37:S9–S19. doi:10.1111/j.1872-034X.2007.00098.x. 17627641

[14] Hughes SA, Wedemeyer H, Harrison PM 2011 Hepatitis delta virus. Lancet 378:73–85. doi:10.1016/S0140-6736(10)61931-9. 21511329

[15] Thursz M, Fontanet A 2014 HCV transmission in industrialized countries and resource-constrained areas. Nat Rev Gastroenterol Hepatol 11:28–35. doi:10.1038/nrgastro.2013.179. 24080775

[16] Andernach IE, Leiss LV, Tarnagda ZS, Tahita MC, Otegbayo JA, Forbi JC, Omilabu S, Gouandjika-Vasilache I, Komas NP, Mbah OP, Muller CP 2014 Characterization of hepatitis delta virus in sub-Saharan Africa. J Clin Microbiol 52:1629–1636. doi:10.1128/JCM.02297-13. 24599979 PMC3993620

[17] Komas NP, Bai-Sepou S, Manirakiza A, Leal J, Bere A, Le Faou A 2010 The prevalence of hepatitis B virus markers in a cohort of students in Bangui, Central African Republic. BMC Infect Dis 10:226. doi:10.1186/1471-2334-10-226. 20670399 PMC2918609

[18] Rao VB, Johari N, du Cros P, Messina J, Ford N, Cooke GS 2015 Hepatitis C seroprevalence and HIV co-infection in sub-Saharan Africa: a systematic review and meta-analysis. Lancet Infect Dis 15:819–824. doi:10.1016/S1473-3099(15)00006-7. 25957078

[19] Winter A, Letang E, Vedastus Kalinjuma A, Kimera N, Ntamatungiro A, Glass T, Moradpour D, Sahli R, Le Gal F, Furrer H, Wandeler G, Group KS 2016 Absence of hepatitis delta infection in a large rural HIV cohort in Tanzania. Int J Infect Dis 46:8–10. doi:10.1016/j.ijid.2016.03.011. 26996457

[20] Makuwa M, Mintsa-Ndong A, Souquiere S, Nkoghe D, Leroy EM, Kazanji M 2009 Prevalence and molecular diversity of hepatitis B virus and hepatitis delta virus in urban and rural populations in northern Gabon in central Africa. J Clin Microbiol 47:2265–2268. doi:10.1128/JCM.02012-08. 19439548 PMC2708505

[21] Kaba M, Colson P, Musongela JP, Tshilolo L, Davoust B 2010 Detection of hepatitis E virus of genotype 3 in a farm pig in Kinshasa (Democratic Republic of the Congo). Infect Genet Evol 10:154–157. doi:10.1016/j.meegid.2009.09.011. 19800029

[22] Werner GT, Frosner GG, Fresenius K 1985 Prevalence of serological hepatitis A and B markers in a rural area of northern Zaire. Am J Trop Med Hyg 34:620–624. 2988353 10.4269/ajtmh.1985.34.620

[23] Laurent C, Henzel D, Mulanga-Kabeya C, Maertens G, Larouze B, Delaporte E 2001 Seroepidemiological survey of hepatitis C virus among commercial sex workers and pregnant women in Kinshasa, Democratic Republic of Congo. Int J Epidemiol 30:872–877. doi:10.1093/ije/30.4.872. 11511619

[24] Batina Agasa S, Dupont E, Kayembe T, Molima P, Malengela R, Kabemba S, Andrien M, Lambermont M, Cotton F, Vertongen F, Gulbis B 2010 Multiple transfusions for sickle cell disease in the Democratic Republic of Congo: the importance of the hepatitis C virus. Transfus Clin Biol 17:254–259. doi:10.1016/j.tracli.2010.09.002. 20961788

[25] Kabinda JM, Miyanga SA, Misingi P, Ramazani SY 2014 Hepatitis B and C among volunteer non-remunerated blood donors in Eastern Democratic Republic of Congo. Transfus Clin Biol 21:111–115. (*In* French.) doi:10.1016/j.tracli.2014.04.001. 24931184

[26] Olivero A, Smedile A 2012 Hepatitis delta virus diagnosis. Semin Liver Dis 32:220–227. doi:10.1055/s-0032-1323627. 22932970

[27] Zhang YY, Tsega E, Hansson BG 1996 Phylogenetic analysis of hepatitis D viruses indicating a new genotype I subgroup among African isolates. J Clin Microbiol 34:3023–3030. 8940442 10.1128/jcm.34.12.3023-3030.1996 PMC229453

[28] Mbendi Nlombi C, Longo-Mbenza B, Mbendi Nsukini S, Muyembe Tamfum JJ, Situakibanza Nanituma H, Vangu Ngoma D 2001 Prevalence of HIV and HBs antigen in blood donors. Residual risk of contamination in blood recipients in East Kinshasa, Democratic Republic of the Congo. Med Trop (Mars) 61:139–142. (*In* French.) 11582869

[29] Kabamba NM, Bwana KI, Kilolo NUE, Kalonji CD, Kabyla IB, Luboya NO 2015 HIV and HBV seroprevalence in volunteer blood donors in Lubumbashi. SOJ Immunol 3(5):1–3. doi:10.15226/2372-0948/3/5/00141.

[30] Batina A, Kabemba S, Malengela R 2007 Infectious markers among blood donors in Democratic Republic of Congo (DRC). Rev Med Brux 28:145–149. (*In* French.) 17708468

[31] Francois-Souquiere S, Makuwa M, Bisvigou U, Kazanji M 2016 Epidemiological and molecular features of hepatitis B and hepatitis delta virus transmission in a remote rural community in central Africa. Infect Genet Evol 39:12–21. doi:10.1016/j.meegid.2015.12.021. 26747245

[32] Mulders MN, Venard V, Njayou M, Edorh AP, Bola Oyefolu AO, Kehinde MO, Muyembe Tamfum JJ, Nebie YK, Maiga I, Ammerlaan W, Fack F, Omilabu SA, Le Faou A, Muller CP 2004 Low genetic diversity despite hyperendemicity of hepatitis B virus genotype E throughout West Africa. J Infect Dis 190:400–408. doi:10.1086/421502. 15216479

[33] Valente F, Lago BV, Castro CA, Almeida AJ, Gomes SA, Soares CC 2010 Epidemiology and molecular characterization of hepatitis B virus in Luanda, Angola. Mem Inst Oswaldo Cruz 105:970–977. doi:10.1590/S0074-02762010000800004. 21225192

[34] Yousif M, Mudawi H, Bakhiet S, Glebe D, Kramvis A 2013 Molecular characterization of hepatitis B virus in liver disease patients and asymptomatic carriers of the virus in Sudan. BMC Infect Dis 13:328. doi:10.1186/1471-2334-13-328. 23865777 PMC3722059

[35] Bekondi C, Olinger CM, Boua N, Talarmin A, Muller CP, Le Faou A, Venard V 2007 Central African Republic is part of the West-African hepatitis B virus genotype E crescent. J Clin Virol 40:31–37. doi:10.1016/j.jcv.2007.05.009. 17689139

[36] Zirabamuzale JT, Opio CK, Bwanga F, Seremba E, Apica BS, Colebunders R, Ocama P 2016 Hepatitis B virus genotypes A and D in Uganda. J Virus Erad 2:19–21. 27482430 10.1016/S2055-6640(20)30693-2 PMC4946690

[37] Mahgoub S, Candotti D, El Ekiaby M, Allain JP 2011 Hepatitis B virus (HBV) infection and recombination between HBV genotypes D and E in asymptomatic blood donors from Khartoum, Sudan. J Clin Microbiol 49:298–306. doi:10.1128/JCM.00867-10. 21048009 PMC3020474

[38] Makuwa M, Souquiere S, Telfer P, Apetrei C, Vray M, Bedjabaga I, Mouinga-Ondeme A, Onanga R, Marx PA, Kazanji M, Roques P, Simon F 2006 Identification of hepatitis B virus subgenotype A3 in rural Gabon. J Med Virol 78:1175–1184. doi:10.1002/jmv.20678. 16847965

[39] Kurbanov F, Tanaka Y, Fujiwara K, Sugauchi F, Mbanya D, Zekeng L, Ndembi N, Ngansop C, Kaptue L, Miura T, Ido E, Hayami M, Ichimura H, Mizokami M 2005 A new subtype (subgenotype) Ac (A3) of hepatitis B virus and recombination between genotypes A and E in Cameroon. J Gen Virol 86:2047–2056. doi:10.1099/vir.0.80922-0. 15958684

[40] Ochwoto M, Kimotho JH, Oyugi J, Okoth F, Kioko H, Mining S, Budambula NL, Giles E, Andonov A, Songok E, Osiowy C 2016 Hepatitis B infection is highly prevalent among patients presenting with jaundice in Kenya. BMC Infect Dis 16:101. doi:10.1186/s12879-016-1409-2. 26932656 PMC4774020

[41] Bekondi C, Mobima T, Ouavene JO, Koffi B, Konamna X, Bere A, Le Faou A 2010 Etiopathological factors of hepatocellular carcinoma in Bangui, Central African Republic: clinical, biological characteristics and virological aspects of patients. Pathol Biol (Paris) 58:152–155. (*In* French.) doi:10.1016/j.patbio.2009.07.027. 19875248

[42] de Lalla F, Rizzardini G, Rinaldi E, Santoro D, Zeli PL, Verga G 1990 HIV, HBV, delta-agent and Treponema pallidum infections in two rural African areas. Trans R Soc Trop Med Hyg 84:144–147. doi:10.1016/0035-9203(90)90412-8. 2189236

[43] Grabowski J, Wedemeyer H 2010 Hepatitis delta: immunopathogenesis and clinical challenges. Dig Dis 28:133–138. doi:10.1159/000282076. 20460901

[44] Makuwa M, Caron M, Souquiere S, Malonga-Mouelet G, Mahe A, Kazanji M 2008 Prevalence and genetic diversity of hepatitis B and delta viruses in pregnant women in Gabon: molecular evidence that hepatitis delta virus clade 8 originates from and is endemic in central Africa. J Clin Microbiol 46:754–756. doi:10.1128/JCM.02142-07. 18077651 PMC2238085

[45] Foupouapouognigni Y, Noah DN, Sartre MT, Njouom R 2011 High prevalence and predominance of hepatitis delta virus genotype 1 infection in Cameroon. J Clin Microbiol 49:1162–1164. doi:10.1128/JCM.01822-10. 21209162 PMC3067734

[46] Greenfield C, Farci P, Osidiana V, Macpherson CN, Romig T, Zeyhle E, French M, Johnson B, Tukei P, Wankya BM, Thomas HC 1986 Hepatitis delta virus infection in Kenya. Its geographic and tribal distribution. Am J Epidemiol 123:416–423. 3946387 10.1093/oxfordjournals.aje.a114256

[47] Ndong-Atome GR, Makuwa M, Njouom R, Branger M, Brun-Vezinet F, Mahe A, Rousset D, Kazanji M 2008 Hepatitis C virus prevalence and genetic diversity among pregnant women in Gabon, central Africa. BMC Infect Dis 8:82. doi:10.1186/1471-2334-8-82. 18559087 PMC2442078

[48] Muzembo BA, Akita T, Matsuoka T, Tanaka J 2016 Systematic review and meta-analysis of hepatitis C virus infection in the Democratic Republic of Congo. Public Health 139:13–21. doi:10.1016/j.puhe.2016.06.017. 27450441

[49] Kamal SM 2008 Acute hepatitis C: a systematic review. Am J Gastroenterol 103:1283–1297. doi:10.1111/j.1572-0241.2008.01825.x. 18477352

[50] Iles JC, Raghwani J, Harrison GL, Pepin J, Djoko CF, Tamoufe U, LeBreton M, Schneider BS, Fair JN, Tshala FM, Kayembe PK, Muyembe JJ, Edidi-Basepeo S, Wolfe ND, Simmonds P, Klenerman P, Pybus OG 2014 Phylogeography and epidemic history of hepatitis C virus genotype 4 in Africa. Virology 464–465:233–243. doi:10.1016/j.virol.2014.07.006. PMC4162651 25105489

[51] Drosten C, Gottig S, Schilling S, Asper M, Panning M, Schmitz H, Gunther S 2002 Rapid detection and quantification of RNA of Ebola and Marburg viruses, Lassa virus, Crimean-Congo hemorrhagic fever virus, Rift Valley fever virus, dengue virus, and yellow fever virus by real-time reverse transcription-PCR. J Clin Microbiol 40:2323–2330. doi:10.1128/JCM.40.7.2323-2330.2002. 12089242 PMC120575

[52] Bivigou-Mboumba B, Francois-Souquiere S, Deleplancque L, Sica J, Mouinga-Ondeme A, Amougou-Atsama M, Chaix ML, Njouom R, Rouet F 2016 Broad range of hepatitis B virus (HBV) patterns, dual circulation of quasi-subgenotype A3 and HBV/E and heterogeneous HBV mutations in HIV-positive patients in Gabon. PLoS One 11:e0143869. doi:10.1371/journal.pone.0143869. 26764909 PMC4713159

[53] Le Gal F, Gordien E, Affolabi D, Hanslik T, Alloui C, Deny P, Gault E 2005 Quantification of hepatitis delta virus RNA in serum by consensus real-time PCR indicates different patterns of virological response to interferon therapy in chronically infected patients. J Clin Microbiol 43:2363–2369. doi:10.1128/JCM.43.5.2363-2369.2005. 15872267 PMC1153793

[54] Kumar R, Pahal V, Singh J 2011 Prevalence of genotype D and precore/core promoter mutations in hepatitis B virus-infected population of North India. J Clin Exp Hepatol 1:73–76. doi:10.1016/S0973-6883(11)60125-4. 25755318 PMC3940627

[55] Ivaniushina V, Radjef N, Alexeeva M, Gault E, Semenov S, Salhi M, Kiselev O, Deny P 2001 Hepatitis delta virus genotypes I and II cocirculate in an endemic area of Yakutia, Russia. J Gen Virol 82:2709–2718. doi:10.1099/0022-1317-82-11-2709. 11602783

[56] Le Gal F, Gault E, Ripault MP, Serpaggi J, Trinchet JC, Gordien E, Deny P 2006 Eighth major clade for hepatitis delta virus. Emerg Infect Dis 12:1447–1450. doi:10.3201/eid1209.060112. 17073101 PMC3294742

[57] Tamura K, Peterson D, Peterson N, Stecher G, Nei M, Kumar S 2011 MEGA5: molecular evolutionary genetics analysis using maximum likelihood, evolutionary distance, and maximum parsimony methods. Mol Biol Evol 28:2731–2739. doi:10.1093/molbev/msr121. 21546353 PMC3203626

