# Supplementary material for: High Prevalence and Diversity of Hepatitis Viruses in Suspected Cases of Yellow Fever in the Democratic Republic of Congo
Source: J Clin Microbiol. 2017 Apr 25;55(5):1299–312. doi: 10.1128/JCM.01847-16 (PMC5405249; doi:10.1128/JCM.01847-16)
Supplement: Supplemental material [file supp_55_5_1299__index.html]

High Prevalence and Diversity of Hepatitis Viruses in Suspected Cases of Yellow Fever in the Democratic Republic of Congo — Supplemental material 

# High Prevalence and Diversity of Hepatitis Viruses in Suspected Cases of Yellow Fever in the Democratic Republic of Congo

## Supplemental material

- Supplemental file 1 -

  Fig. S1 (Prevalence of hepatitis viruses among patients with jaundice who tested negative for yellow fever in each province of the Democratic Republic of Congo. Black bars show the percentage of patients infected with at least one hepatitis virus in each province. Letters above the error bars show the location of the province: W, western; N, northern; C, central; S, southern; E, eastern).

  PDF, 606K
