# Supplementary figures and images for: High Prevalence and Diversity of Hepatitis Viruses in Suspected Cases of Yellow Fever in the Democratic Republic of Congo
Source: J Clin Microbiol. 2017 Apr 25;55(5):1299–312. doi: 10.1128/JCM.01847-16 (PMC5405249; doi:10.1128/JCM.01847-16)

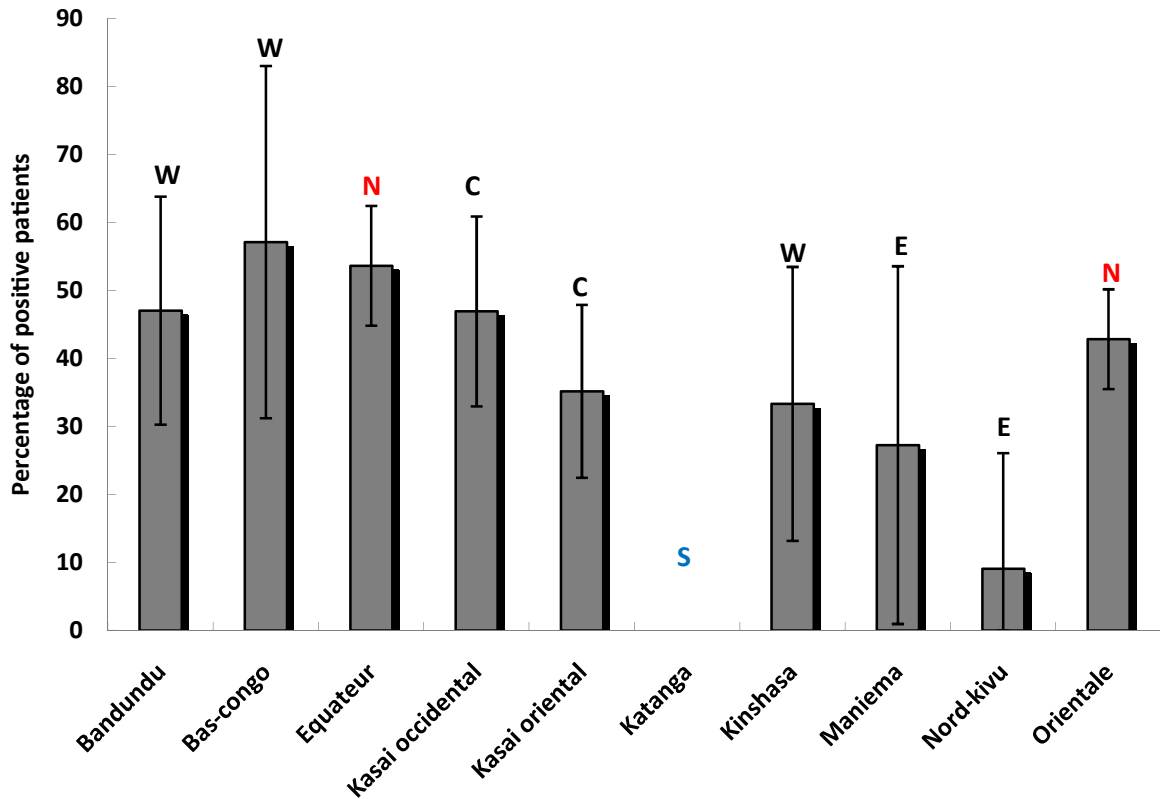

Supplement: Supplemental material [file JCM.01847-16_zjm999095446s1.pdf]
